# Neuroendocrine plasticity and crosstalk in pubertal development

**DOI:** 10.1111/jne.70145

**Published:** 2026-02-19

**Authors:** Carol Fuzeti Elias, Xingfa Han, David Garcia‐Galiano, Cristina Sáenz de Miera

**Affiliations:** ^1^ Department of Molecular & Integrative Physiology University of Michigan Ann Arbor Michigan USA; ^2^ Department of Obstetrics and Gynecology University of Michigan Ann Arbor Michigan USA; ^3^ Department of Bioengineering and Applied Biology Sichuan Agricultural University Ya'an China; ^4^ Instituto Maimónides de Investigación Biomédica de Córdoba (IMIBIC) Córdoba Spain; ^5^ Department of Cell Biology, Physiology and Immunology University of Córdoba Córdoba Spain

**Keywords:** dopamine transporter, growth hormone, kisspeptin, obesity, reproduction

## Abstract

Puberty is a critical developmental stage during which individuals acquire the capacity for sexual reproduction. This transition involves a series of complex biological events primarily orchestrated by the activation of the hypothalamo–pituitary–gonadal (HPG) axis. Central to this process are gonadotropin‐releasing hormone (GnRH) neurons, which play a key role in regulating reproductive maturation and function throughout life. However, the precise mechanisms that trigger the pubertal increase in GnRH activity remain incompletely understood. Evidence from our laboratory indicates that a profound remodeling of the hypothalamus is crucial for sexual maturation. In this review, we discuss findings from our research utilizing a combination of RNA sequencing, conditional genetic manipulation with mouse models and viral vectors, and systems neuroscience approaches. Our results reveal that the pubertal transition involves changes in the chemical phenotype and site‐specific innervation of key hypothalamic neurons. Among these neuronal populations, those expressing growth hormone‐releasing hormone (GHRH), kisspeptin, or dopamine transporter (DAT) are the focus of this review. Building upon data from other laboratories, our findings offer new insights into the neural and molecular mechanisms by which the hypothalamus orchestrates sexual maturation.

## 1 INTRODUCTION

Puberty is the critical developmental stage during which individuals gain the capacity for sexual reproduction. This transition involves a series of intricate biological events coordinated primarily by the activation of the hypothalamo–pituitary–gonadal (HPG) axis.^1^, ^2^, ^3^ Central to this process are the gonadotropin‐releasing hormone (GnRH) neurons, which serve as the crucial regulators of reproductive maturation and function throughout life.

The pulsatile secretion of GnRH prompts the anterior pituitary gland to synthesize and release gonadotropins, that is, the luteinizing hormone (LH) and the follicle stimulating hormone (FSH). Elevated gonadotropin pulsatile release then drives the maturation of gonadal tissues, the production of sex steroids, and the maturation of the gametes.

While the activation of GnRH neurons is essential for reproductive development, the precise triggers leading to the increase in GnRH activity at puberty remain incompletely understood. Factors such as neuronal input plasticity, epigenetic modifications, or structural remodeling may contribute, but their roles as initiating factors are still under investigation.^1^, ^4^, ^5^, ^6^ Among the last, nutrition emerges as a crucial factor in pubertal timing.

The central nervous system (CNS) integrates signals related to energy stores and nutritional status with reproductive function.^7^ These metabolic signals include both macronutrients like glucose, amino acids, and fatty acids and hormones released by metabolic organs, such as white adipocyte‐derived leptin, pancreatic insulin and glucagon, and gut peptides. Many of these hormones act directly on hypothalamic circuits affecting GnRH neuronal activity, thereby coordinating reproductive function with the metabolic state.

One of our research interests is to define the neural and molecular basis of the metabolic control of the reproductive function, including pubertal development. Our main focus has been on the neural effects of leptin in reproduction because loss‐of‐function mutations that impair either leptin or its cognate receptor (LepRb) result in severe metabolic disturbances and lack of pubertal development leading to infertility in both rodents and humans.^4^, ^8^, ^9^, ^10^, ^11^

In leptin‐deficient mice (*ob/ob, Lep* ^*ob*^), the distribution of GnRH neurons in the brain is unaffected, but the density of their terminals in the median eminence is elevated whereas gonadotropin levels in circulation are reduced.^12^, ^13^, ^14^ Leptin administration to leptin‐deficient mice and humans normalizes body weight, promotes sexual maturation and restores fertility.^10^, ^15^, ^16^ It is important to stress that the effects of leptin on pubertal development are only achieved in individuals at the adequate developmental stage, that is, timely growth and organs differentiation.^9^ Notably, administering leptin to wild‐type prepubertal females at low doses that do not affect body weight accelerates pubertal maturation.^17^, ^18^, ^19^

Studies from various laboratories have demonstrated that leptin acts within the hypothalamus on neurons upstream of GnRH neurons inducing plastic changes that stimulate GnRH secretion. Thus, understanding the development of neuroendocrine systems that regulate metabolism will generate insights into the events necessary for typical pubertal development. In this review, we focus on two highly relevant hypothalamic nuclei: the arcuate nucleus (ARH) and the ventral premammillary nucleus (PMv).^4^, ^7^

## 2 INSIGHTS FROM COMBINED RNA SEQUENCING

Pubertal development is a continuous process defined by differentiation and growth in a time‐dependent manner. In mice and rats, vaginal opening is a consensus marker of puberty onset, and the first estrus defines puberty completion. Whereas puberty completion can be timely monitored, the events associated with puberty onset are difficult to identify. Several laboratories have proposed that in mice, changes in hypothalamic transcriptome occur several days before vaginal opening.^20^ The exact timing, however, varies among individuals of the same mouse line and genetic background.

To gain insights into the genes associated with hypothalamic actions on puberty onset, we took advantage of the infertile prepubertal *Lep* ^*ob*^ mouse, in which we can induce puberty with leptin administration and thus control the timing of puberty onset. Because the *Lep* ^*ob*^ mice have a series of metabolic and neuroendocrine dysregulation, we compared the data obtained from *Lep* ^*ob*^ with those from prepubertal and adult C57BL6/J females. We chose to focus on females because puberty onset and completion are easily monitored via analysis of vaginal opening and cellular makeup of vaginal lavage.^21^

In previous studies, we observed that 2 days of leptin treatment induces vaginal opening in *Lep* ^*ob*^ female mice.^13^ Using this approach, we evaluated the transcriptomic changes of hypothalamic sites before and after the puberty‐inducing leptin treatment using two strategies: (a) Bulk RNA‐sequencing of micro dissected mediobasal hypothalamus, and (b) Translating ribosome affinity purification (TRAP)‐sequencing of leptin‐responsive cells using micro punches of the ARH and PMv of LepRb‐Cre L10 *Lep* ^*ob*^ female mice.^21^, ^22^, ^23^ In both approaches, we compared groups of 8‐week‐old *Lep* ^*ob*^ female mice treated with saline with those treated with leptin in 48 h (4 injections). Saline treated mice showed no vaginal opening and were used as negative controls. These mice provided relevant data on gene expression in mediobasal hypothalamus and in ARH and PMv LepRb neurons of a prepubertal female. Leptin treated mice showed vaginal opening and were used to determine leptin‐induced changes in gene expression in mediobasal hypothalamus and specifically in ARH and PMv LepRb neurons that are potentially involved with the activation of the reproductive neuroendocrine axis and sexual maturation.^21^, ^24^

**FIGURE 1:**
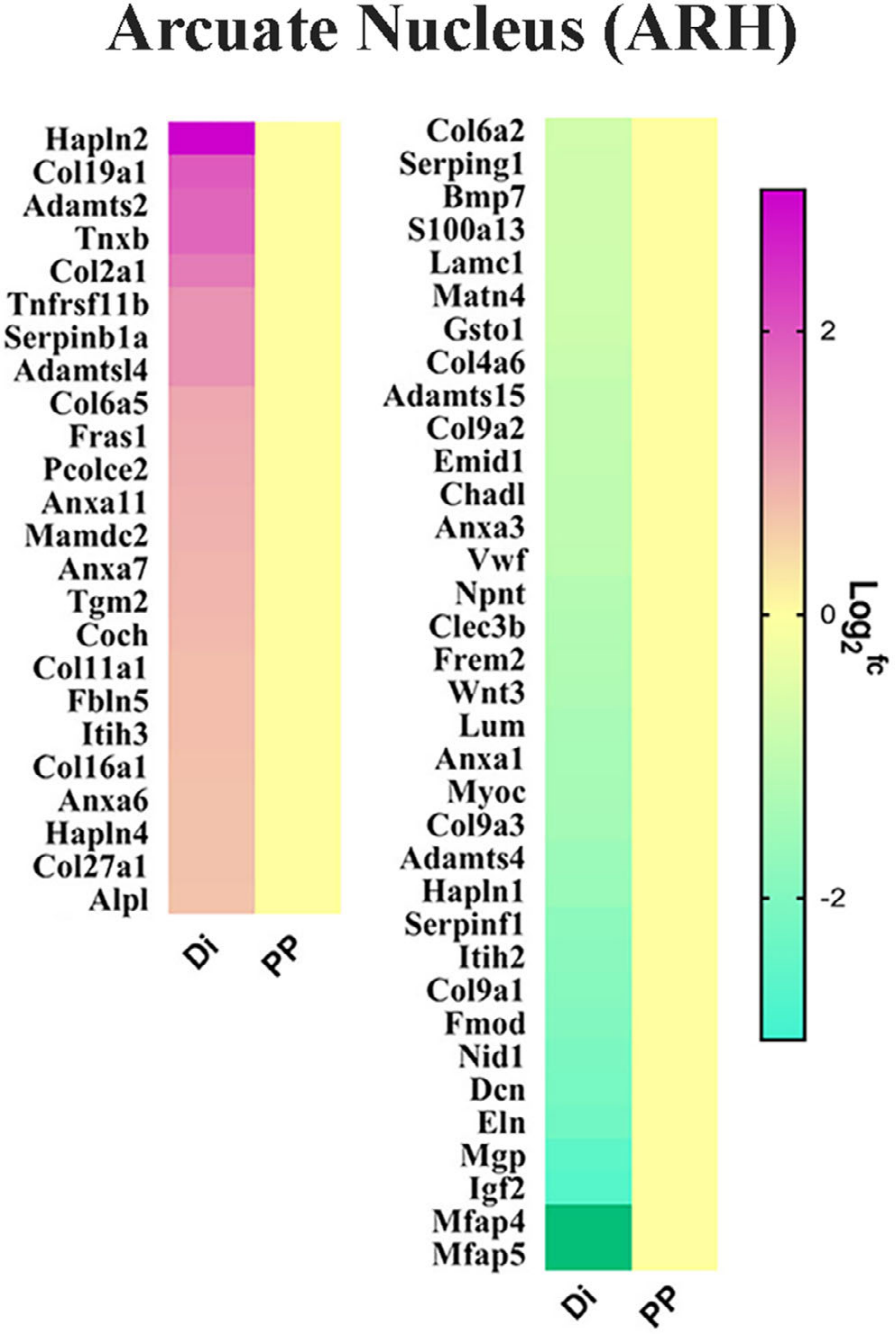
Differentially expressed genes (DEGs) associated with extracellular matrix in arcuate nucleus (ARH) of prepubertal (PP) vs. diestrous (Di) female mice. Data obtained from Han et al., 2020.^21^ DEGs in the PMv were published elsewhere.^24^

An additional group consisting of prepubertal and age‐matched diestrous C57BL6/J mice treated with saline was used as positive control. In those mice, ARH and PMv micro punches were collected and subjected to RNA sequencing as well. This approach provided a comprehensive profile of gene expression in prepubertal and adult ARH and PMv cells.^21^ Through overlapping analyses of those RNA‐seq data, we identified a series of LepRb enriched and differentially expressed genes (DEGs) in the ARH and PMv associated with leptin‐induced and typical pubertal progression.^21^ These data highlighted a number of potential neuroendocrine and neurochemical players on puberty onset, including a number of pathways related to structural remodeling and plasticity (Figure 1).

The DEGs were mostly upregulated in the ARH and downregulated in the PMv of prepubertal mice. This is noteworthy as LepRb neurons in the ARH and PMv associated with reproductive control respond to leptin in opposite directions.^13^, ^25^, ^26^, ^27^, ^28^ In the ARH, leptin inhibits GABAergic agouti‐related peptide (AgRP) neurons, which in turn inhibit kisspeptin neurons. Leptin would then induce a disinhibitory effect on ARH kisspeptin neurons via inhibition of AgRP/NPY. In contrast, leptin mostly depolarizes (activates) glutamatergic PMv neurons, which then stimulate the reproductive axis by acting directly on GnRH terminals or on kisspeptin neurons.^13^, ^27^, ^29^

In the following sections, we will discuss two of those DEGs: the growth hormone releasing hormone (*Ghrh*) in the ARH and the dopamine transporter or solute carrier family 6, member 3 (*Slc6a3*) in the PMv. We will focus on research from our laboratory showing reorganization of these neuronal populations during pubertal development.

## 3 GROWTH HORMONE RELEASING HORMONE (GHRH): THE USUAL SUSPECT

Circulating growth hormone (GH) concentration in humans and mice rises at birth, decreases during infancy, and increases again at puberty when it promotes growth spurt. Another usually overlooked action of GH is on sexual maturation. Individuals with disrupted somatotropic (growth) axis also show delayed puberty.^30^, ^31^, ^32^

*Ghrh* expression is enriched in ARH LepRb neurons and increases during pubertal maturation.^33^, ^34^ Mice with lack of leptin signaling (LepRb) in GHRH neurons show no difference in body weight, fat and lean mass, food intake, glucose homeostasis or body length compared to controls. The reproductive phenotype of those mice, however, including pubertal timing, was not reported.^34^

The pubertal increase in GH secretion is thought to be a direct response to gonadal maturation and the increase in circulating levels of gonadal steroids after a period of quiescence of the HPG axis. In a recent publication, we assessed if direct estrogen signaling via ERα in GHRH neurons is necessary for pubertal maturation and growth.^33^

Mice with deletion of ERα in GHRH (GHRH^*ΔEsr1*^) neurons showed decreased hypothalamic *Ghrh* expression and low insulin‐like growth factor 1 (IGF‐1) production. As a result, these mice were shorter due to delayed epiphyseal fusion. Puberty onset was not altered but puberty completion (first estrus) was delayed.^33^

To further explore the mechanisms underlying the role of GHRH neurons in pubertal progression, we assessed their potential interaction with neighboring neurons crucial for sexual maturation in humans and rodents. Kisspeptins, encoded by the *Kiss1* gene, are key mediators of estrogen feedback that regulate pubertal timing and fertility.^35^, ^36^ Nearly all ARH Kiss1 neurons express ERα at various developmental stages.^37^, ^38^, ^39^

During pubertal transition, *Kiss1* gene and the number of neurons expressing Kiss1‐Cre induced reporter gene (eGFP) are increased in the ARH.^33^, ^40^ After pubertal growth spurt, *GHRH* gene expression in the ARH decreases in association with the gradual decline in GH secretion. Using Cre‐induced reporter genes (L10‐eGFP), we observed, however, that the number of GHRH‐Cre eGFP neurons is higher in adult compared to prepubertal mice. As gonadal steroid synthesis persists beyond puberty, we propose that a transition occurs in the expression and secretion patterns of GHRH and Kiss1 during pubertal development. Supporting this view, studies have shown that kisspeptins inhibit GH release independently of direct GH feedback mechanisms.^41^, ^42^, ^43^ Thus, an increase in kisspeptin production at the expense of GHRH release is expected to reduce GH secretion and potentially activate the HPG axis.

In adult female mice, but not in males, approximately half of Kiss1‐hrGFP neurons coexpress GHRH^Cre^‐tdTom. Because prepubertal mice show virtually no colocalization of both reporter genes, and higher numbers of Kiss1^Cre^‐ or GHRH^Cre^‐eGFP neurons are observed during pubertal maturation, we hypothesized that Kiss1/GHRH observed colocalization is the effect of gonadal hormone's action in the expression of both neuropeptides in overlapping neurons during pubertal transition. Following puberty completion, *Ghrh* decreases with the end of the growth spurt whereas *Kiss1* expression increases exerting a key role in GnRH pulsatile release and estrous cycle. Even though reporter genes indicate that a subpopulation of Kiss1 neurons also release GHRH, coexpression of *Ghrh* and *Kiss1* genes is negligible in adult females. Considering that the Cre‐induced reporter gene is a tool for cell lineage tracing or gene expression during development, we propose that a subpopulation of dual phenotype ARH neurons produce GHRH during the pubertal growth spurt and become kisspeptinergic once sexual maturation is completed.^33^

**FIGURE 2:**
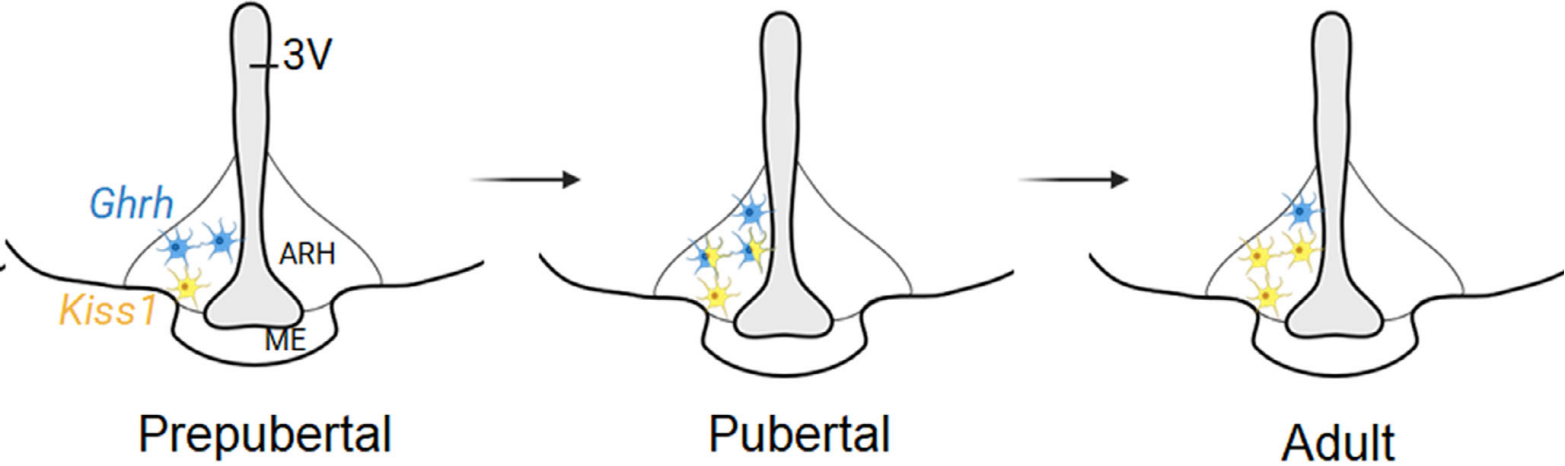
Model for plasticity of *Ghrh* and *Kiss1* cells in arcuate nucleus (ARH) during the pubertal transition in females. ME, median eminence; 3V, third ventricle. Figure prepared using Biorender.

This potential shift in neuronal phenotype might be a key event in hypothalamic remodeling from an immature to an adult structure (Figure 2). It would represent a coordinated crosstalk between the growth and the reproductive axes during the critical time of puberty. Additional studies, however, are necessary to test this model.

## 4 DOPAMINE TRANSPORTER (DAT): AN UNEXPECTED CONTENDER

Following the combined RNA sequencing analysis, several DEGs components of the dopaminergic system were observed in protein–protein interaction and network modeling of both ARH and PMv. Of note, *Nr4a2* (*Nurr1*), *Cdkn1c*, *Ddc*, *Th*, *Gpr88*, *Drd3*, and *Slc6a3* were highly represented in our database.^21^

The tubero‐infundibular dopamine (TIDA) neuronal subpopulation of ARH neurons is a well‐described player in the control of pituitary prolactin release,^44^ but the PMv neurons expressing dopamine transporters (DAT) are less characterized. Several groups have shown that a subset of PMv neurons expresses the dopamine transporter (DAT, *Slc6a3* gene), a presynaptic transporter involved with dopamine reuptake from the synaptic cleft.^45^, ^46^, ^47^ The PMv DAT neurons, however, are unique as they show undetectable levels of tyrosine hydroxylase (TH), the rate‐limiting enzyme for catecholamines production.^46^, ^48^ This population has been explored for its role in social behaviors, inter‐male and maternal aggression.^46^, ^47^, ^49^, ^50^

**FIGURE 3:**
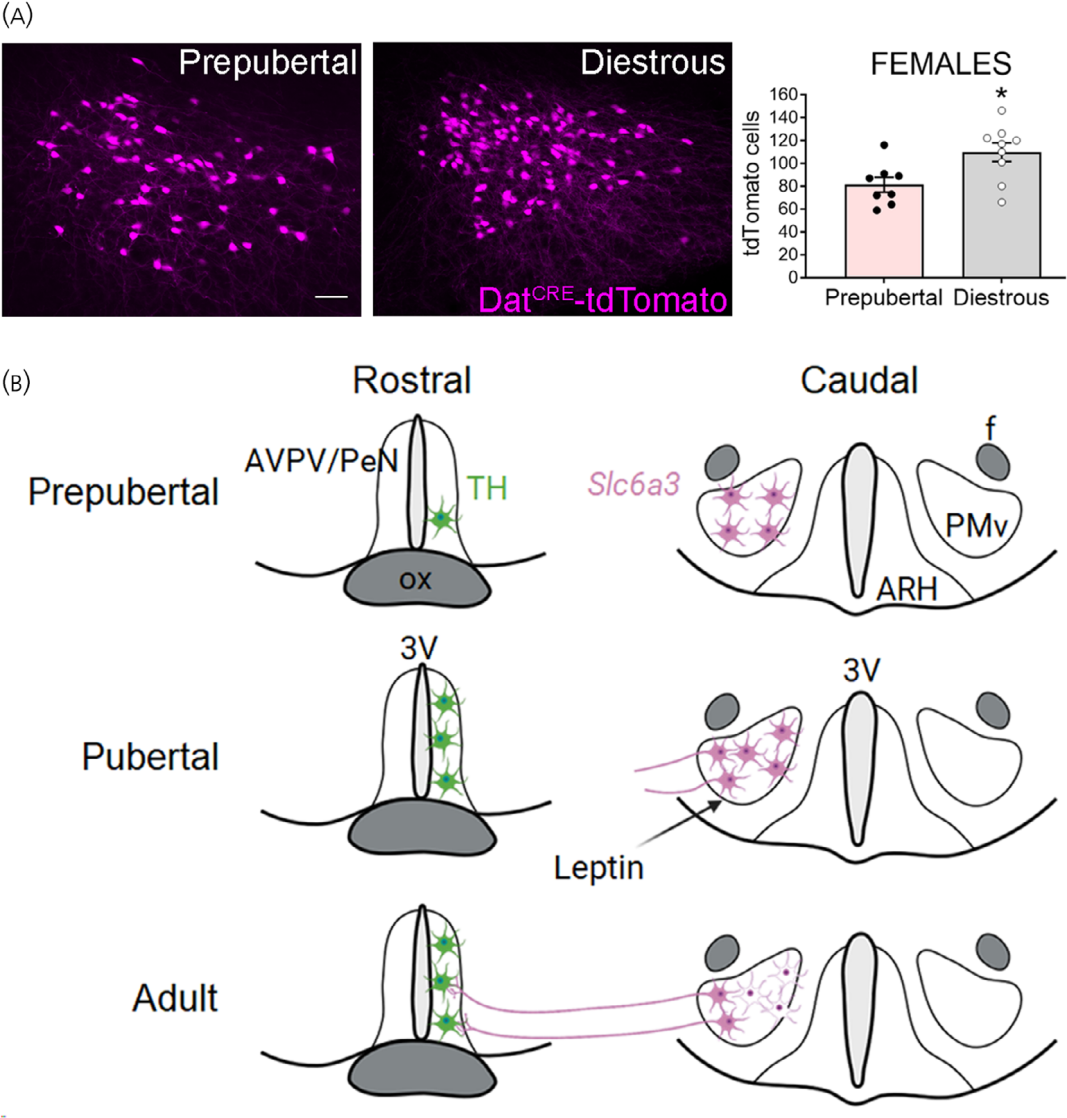
Model for chemical plasticity and innervation remodeling of leptin‐sensitive *Slc6a3* neurons in PMv during pubertal transition in female mouse. (A) Number of tdTomato cells per section in the ventral premammillary nucleus (PMv) of prepubertal (*n* = 8) and adult females in diestrous (*n* = 9). Unpaired *t*‐test; *t* (15) = 2.686, *p* = .017. Experimental and analysis details can be found in Sáenz de Miera et al., 2025.^51^ (B) Model. AVPV/PeN, anteroventral periventricular nucleus and periventricular nucleus; 3V, third ventricle; ARH, arcuate nucleus; TH, tyrosine hydroxylase; ox, optic chiasm; f, fornix. Panel B prepared using Biorender.

We showed that *Slc6a3* (*DAT*) mRNA expression in the PMv is higher in females and in prepubertal mice. However, we observed a higher number of DAT^Cre^‐tdTomato neurons in adult females (*p* = .017, unpaired *t*‐test), suggesting an upregulation during sexual maturation (Figure 3A). Changing levels of estradiol is not the cause of this regulation since ovariectomy and estrogen replacement had no obvious effect on *Slc6a3* mRNA levels.^51^

*Slc6a3* is expressed in a subpopulation of LepRb neurons of the PMv. Juvenile overnutrition caused by rearing mice in a small litter size^52^, ^53^ induces early puberty and increases levels of *Slc6a3* mRNA expression in PMv in females, correlated with the individual's body mass. Furthermore, PMv *Slc6a3* mRNA levels are decreased in *Lep* ^*ob*^ mice and are elevated by leptin treatment, suggesting that leptin works as a signal to increase *Slc6a3* gene expression during pubertal transition. These findings indicate that PMv DAT neurons are responsive to nutritional cues.

Since DAT is mostly found in presynaptic terminals,^54^ PMV DAT neurons may have a role regulating dopamine availability produced by dopaminergic TH‐positive neurons at the synaptic cleft. Following reuptake by DAT, dopamine may be recycled and become available for subsequent release.^55^, ^56^, ^57^ In fact, we found that PMv DAT neurons project to and make apparent synaptic contacts with kisspeptin and TH neurons of the anteroventral periventricular nucleus and periventricular nucleus^51^ (AVPV/PeN, aka RP3V^58^). This projection is observed only in adult, not prepubertal, female mice, being established during pubertal transition concomitant with a sharp increase in AVPV/PeN *Kiss1* and *Th* gene expression.^51^, ^59^, ^60^, ^61^, ^62^, ^63^

Together, our findings suggest that the PMv DAT neuronal population conveys signals from nutritional state to AVPV/PeN Kiss1 and TH neurons potentially modulating dopamine microcircuitry in brain sites relevant for female ovulation,^64^ a model that needs to be tested. These dynamic changes in *Slc6a3* expression and neuronal projections suggest that the PMv DAT population undergoes chemical and innervation remodeling during pubertal maturation (Figure 3B), contributing to the emergence of adult reproductive function.

## 5 CONCLUSION

The studies discussed in this review expand on the proposed model that microstructural changes within the neuroendocrine hypothalamus are essential for typical and timely pubertal development. Our data indicate that both a shift in the chemical phenotype of GHRH/Kiss1 neurons in the ARH^33^ as well as changes in PMv DAT innervation of AVPV Kiss1 and TH neurons^51^ play key roles in these processes.

Supporting the importance of hypothalamic remodeling during puberty are findings related to makorin ring finger protein 3 (MKRN3), a key inhibitor of puberty onset.^65^ Experimental studies in mice demonstrated that hypothalamic *Mkrn3* decreases at pubertal stages and deletion of *Mkrn3* results in early puberty completion with increased density of dendritic spines within the ARH,^66^ suggesting that MKRN3 modulates puberty onset, at least in part, by influencing hypothalamic remodeling. While the models are not directly comparable, our transcriptomic data confirmed that *Mkrn3* expression is elevated in the ARH and PMv of prepubertal compared to adult female mice.^21^ We did not detect enriched expression in LepRb neurons, indicating that MKRN3's effects on pubertal timing are mediated through pathways outside of leptin‐responsive neurons that are still undefined.^21^, ^67^

It is important to emphasize that puberty is a complex process, requiring the coordinated actions of hormones, growth factors, genetic and epigenetic components, and tissue remodeling within a precise timeframe.^3^, ^68^, ^69^, ^70^, ^71^, ^72^, ^73^, ^74^, ^75^ Comprehensive reviews on the role of hormones, peptides, and receptors, including other components of the melanocortin system such as POMC, MC3R, and MC4R, have been published and may be consulted for a better understanding of other factors involved in the transition from childhood to sexual maturity.^4^, ^11^

## AUTHOR CONTRIBUTIONS

**Carol Fuzeti Elias:** Writing – review and editing; writing – original draft; funding acquisition; conceptualization; supervision. **Xingfa Han:** Writing – review and editing. **David Garcia‐Galiano:** Writing – review and editing. **Cristina Sáenz de Miera:** Writing – review and editing; writing – original draft; visualization; conceptualization.

## CONFLICT OF INTEREST STATEMENT

The authors declare no conflicts of interest.

## Data Availability

Data sharing not applicable to this article as no datasets were generated or analyzed during the current study.
